# Public knowledge and attitudes towards Human Papilloma Virus (HPV) vaccination

**DOI:** 10.1186/1471-2458-8-368

**Published:** 2008-10-23

**Authors:** Charlotte Devereaux Walsh, Aradhana Gera, Meeraj Shah, Amit Sharma, Judy E Powell, Sue Wilson

**Affiliations:** 1 Department of Primary Care and General Practice, Division of Primary Care, Public and Occupational Health, The University of Birmingham, Birmingham B15 2TT, UK; 2 The Medical School, The University of Birmingham, University of Birmingham, Birmingham B15 2TT, UK; 3 Department of Public Health, Division of Public Health and Epidemiology, The University of Birmingham, Birmingham B15 2TT, UK

## Abstract

**Background:**

Human Papilloma Virus (HPV) vaccine has undergone successful trials and has recently been approved for use for the primary prevention of cervical cancer. The aim of this study was to determine knowledge and attitudes towards HPV vaccination.

**Methods:**

Semi-structured interview and questionnaire delivered in a street survey. Standardised HPV-related statements used to measure HPV knowledge and attitudes to vaccination. The setting was three different areas of Birmingham, to target a mix of social class and ethnicity. The sample population was composed of 16–54 year olds.

**Results:**

A total of 420 participants were recruited. Poor knowledge of HPV and its links with cervical cancer were observed. 81% had a knowledge score of zero. Knowledge about HPV was associated with different ethnic group and socio-economic group. The majority (88%) of participants were in favour of vaccination, with 83.6% indicating that they would allow a child under their care to be vaccinated.

**Conclusion:**

Initial responses to the proposed HPV vaccination within the UK public are favourable. However, knowledge levels are poor and media and health professional promotion are required to raise awareness.

## Background

Since the 1970's it has been known that one of the causes of cervical cancer is through infection with Human Papilloma Virus (HPV) [1] subtypes of HPV causing malignant transformation of the cervical epithelium [1,2]. This breakthrough led to the possibility that some cervical cancers could be preventable by vaccination [1,2]. There are approximately one hundred different genotypes of HPV. [3] Forty of these types infect the genitalia, and fifteen put women at a high risk of cervical cancer [3,4]. In the UK and many other countries, HPV-16 and 18 are the most common subtypes associated with cervical carcinoma and are believed to be implicated in the aetiology of 70% of malignancies [2,3].

An HPV vaccine has been developed by two separate drug companies: Merck, which produces Gardasil, and GlaxoSmithKline, which produces Cervarix [5]. Both drugs have undergone phase III trials, and were approved for use in the USA in 2006, the Merck vaccine was also approved for use in the UK in 2006 [1,6-10]. To maximise effectiveness, the vaccine needs to be given to girls before they are sexually active [11]. Initial discussions for a UK vaccination programme suggested targeting girls aged 10–12 years [12]. On 26^th ^October 2007, the Department of Health (England) announced the Government immunisation programme would commence in 2009 with HPV vaccination of girls aged 12–13 years [13,14].

Despite HPV vaccination being a relatively new concept, there are a number of studies that have discussed the effectiveness of the vaccine and how it works [6-9,15]. However, relatively few investigations have addressed the public's support for vaccinating school age children. The majority of the research that has been undertaken has not been based in the UK [16-20] and reports parental (especially maternal) and adolescent attitudes towards HPV vaccination, rather than the attitudes of the general public [19-22].

A Finnish study reported 86% of parents and adolescents approved of the vaccine [19] and over 90% of mothers in Vietnam were in favour of their daughters receiving the vaccine although only 1% had previous awareness of the possibility of vaccination [20]. Such findings are re-enforced by a recent review of the literature on HPV vaccine acceptability which reported that many women had a poor knowledge about HPV but, nevertheless, most parents are interested in having their children vaccinated [23].

A Canadian study, published in 2000, assessing public knowledge reported that only 13% of adolescents had heard about HPV [24], however a more recent study, conducted 2006–07, reported that 70% of respondents with female children intended to have their daughters vaccinated [25]. A study, in Georgia, undertook 35 focus groups with stratification by gender, race and location. Knowledge, attitude and beliefs about HPV were explored. Low knowledge and the association of the vaccine with STD were concluded to be barriers to future acceptance of the vaccine [26].

The relatively small number of UK based studies undertaken to date also tend to report that people generally know little about HPV, but after the provision of information, most support the introduction of the vaccine [27-30]. A recent survey of British women aged 16–97 used an open question to elicit the causes of cervical cancer; only 2.5% mentioned HPV and 7% mentioned an unspecified sexually transmitted virus/infection [28]. However, the majority of parents of 11–12 year old school children state that they would allow their child to be vaccinated [27,30] and only a minority express concern that it may encourage unsafe sexual behaviour [27,30]. This latter issue has appeared in national newspapers, with one paper leading with the headline "NHS giving out 'promiscuity jab' by the backdoor" [31].

In summary the literature published to date shows low levels of public knowledge about HPV and its links to cervical cancer. However, the majority of studies also show that people are generally in favour of vaccination.

This study aimed to determine public awareness of, and attitudes towards, HPV vaccination in three urban areas of Birmingham. Secondary aims were to determine the effect of social class and ethnicity on attitudes towards the proposed vaccine. A better understanding of factors that may be associated with low knowledge or negative attitudes to screening being important to inform the targeting of future awareness campaigns.

## Methods

### Settings/Study Population

Participants were recruited from the Birmingham area of the United Kingdom during period April-May 2007. The Birmingham conurbation has a 66% White British population, compared with a national average of 87% [32]. Ethnic minority groups (excluding White Irish) make up 29.6% of the population compared with the national average of 7.9%. These ethnic groups (in order of size) comprise Pakistani, Indian, Black Caribbean, mixed races, Bangladeshi, and smaller groups such as Chinese and Black Africans.

To ensure a range of participants were recruited, data collection was conducted in three areas of Birmingham (Northfield – predominantly White, British, Ladywood – relatively high proportion of ethnic minorities, Sutton Coldfield – population generally of a higher social class compared to the other two areas), which were purposively chosen to reflect the diversity of social class and ethnicity across the city.

### Data collection

This cross-sectional survey was conducted as a street survey. Members of the public were recruited to participate in a semi-structured interview using flash cards as prompts for the collection of categorical data. The main street of each area was used to ensure access to a large and varied selection of people. All interviews were conducted by four medical students. Practice interviews were conducted before the commencement of data collection to ensure that interviews were undertaken in a standardised manner.

Purposive sampling was used to ensure the inclusion of sufficient subjects in each sub-group of interest (age, gender, socio-economic group, ethnicity). The composition of the study sample was predetermined and aimed to recruit adults (aged 16–54 years) in the following proportions: white 70%, ethnic minorities 30%; three geographical locations: 33.3% each; non manual workers (social classes 1-3N) 50%, manual workers (social classes 3M-5) 50%; male 50%, female 50%; age bands, 16–24, 25–34, 35–55, 45–54, 25% each).

Balanced groups were obtained by pre-determining the number of people required in each sub-group of interest. After a short introduction about the study, potential participants were first asked their age, then ethnicity and socio-economic group to determine eligibility. If sufficient participants had been recruited to the relevant sub-group, potential participants were thanked for their time and told that sufficient people in this sub-group had already been recruited. Recruitment continued in each of the three geographical areas until sufficient people had been recruited into each strata. Information on the recruitment rate and the characteristics of non-participants were not collected.

Social class was assessed using the Registrar General's Social Scale [33] which has six groups (1: Professional e.g. accountant, Doctor, Lawyer; 2: Intermediate e.g. manager, school teacher, nurse; 3N: Skilled Non-manual e.g. Clerical worker, secretary and shop assistant; 3M: Skilled Manual e.g. Bus driver, Coal-face worker, Carpenter; 4: Semi-skilled manual e.g. Agricultural worker, Bus conductor, Postman; 5: Unskilled manual e.g. Labourer, Cleaner, Dock worker). Ethnicity was assessed using the criteria for the UK 2001 Census [34].

A sample size of 383 was pre-specified as being sufficient to determine the proportion of people with a knowledge score of less than 2 with 5% precision, 90% power and 95% confidence [35].

### Measurement and Analysis

Data collected were entered into SPSS V 14.0. Data entry errors were resolved by reference to data collection sheets.

Questionnaire administration commenced with socio-demographic questions, followed by an assessment of HPV knowledge. Therefore, for each respondent, a knowledge score was calculated from six HPV related questions. One point was allocated for each positive ("yes") answer resulting in a score from zero to six [0 (no knowledge) to 6 (high knowledge)]. If the respondent answered "No" to the first of these questions ("Have you heard of HPV?"), then the remaining HPV knowledge questions were not asked. For the purposes of modelling, the knowledge score was reduced to a binary variable (no knowledge: knowledge score = 0 vs. some knowledge: knowledge score >0).

The six questions asked comprised:

1) Have you heard about Human Papilloma Virus?

2) Did you know the Human Papilloma Virus can be transmitted through sexual intercourse?

3) Have you heard about the HPV vaccination?

4) Did you know it is a vaccination which protects women against cervical cancer?

5) Did you know that the government is considering offering HPV vaccination to school girls aged 10–12?

6) Did you know a lot of women have already acquired one or more HPV type(s) covered by the vaccine?

Subsequent to the provision of standardised information (information provided to all participants, irrespective of HPV knowledge score) relating to HPV, attitudes to HPV vaccination were assessed using the question: *"Do you think that the introduction of HPV vaccination is a good idea?" *A Likert scale ranging from strongly agree (score: 1) to strongly disagree (score: 5) was used. For the purposes of modelling, the attitude score was reduced to a binary variable (negative attitude: no opinion, disagree and strongly disagree, vs. positive attitude: agree and strongly agree). The interview ended with some questions relating to how much people were prepared to pay for vaccination and whether boys should also be vaccinated.

To facilitate modelling, independent variables were dichotomised to: manual (3M-5) vs. non-manual (1-3N), older (age 45+) vs. younger (age <45), white vs. non-white (all ethnic groups other than white British).

A parent was defined as anyone with children regardless of age of the child. Parents were asked to imagine when their child was the target age (10–12 years) and to answer the questions relating to attitudes to vaccination accordingly.

Data were not normally distributed. The Mann Whitney U test (gender, social class, ethnicity, parenthood) and Kruskal-Wallis test (age groups, areas) were used to test for differences between groups in knowledge and attitude scores. Mean knowledge scores are reported in the text, all groups of interest having a median score of zero. Binary logistic regression (forward stepwise method) was conducted, using SPPS v14, to explore the effect of variables demonstrated to be significantly associated with the outcomes of interest (knowledge, attitude) in the univariate analyses.

***Ethical approval ***for this study was provided by South Birmingham Research Ethics Committee in November 2006 (ref: S/2006/113).

## Results

One hundred and forty people in each of the three locations were recruited, generating a total sample of 420 (Table 1). Of the 420 people, 315 (75%) were white and 105 (25%) ethnic minorities. The non-manual group (social classes I – IIIM) comprised 241 (57.4%) people with 179 (42.6%) in the manual (social classes IV – V). 159 (37.9%) participants were male and 261(62.1%) female. There were 20%–30% participants in (each of the four age group bands). Fifty-nine per cent of the 420 participants were parents.

**Table 1:** Characteristics of participants

		**N**	**%**
**Area**			
	Ladywood	140	33.3
	Northfield	140	33.3
	Sutton Coldfield	140	33.3

**Age Group**			
	16–24	110	26.2
	25–34	91	21.7

	35–44	126	30.0
	45–54	93	22.1

**Gender**			
	Male	159	37.9
	Female	261	62.1

**Ethnicity**			
	White British and Irish	305	72.6
	Mixed	13	3.1
	Asian (Indian, Pakistani, Bangladeshi or other)	54	12.9
	Black (Black African, Black Caribbean or other)	37	8.8
	Other (including Chinese)	11	2.6

**Social Class**			
	I	9	2.1
	II	67	16.0
	IIIN	109	26.0
	IIIM	56	13.3
	IV	61	14.5
	V	118	28.1

**Parents**			
	Yes	248	59.0
	No	172	41.0

### Knowledge Score

Three hundred and forty (81%, 95% CI = 76.9% to 84.6%) of the study participants had a knowledge score of 0 (mean = 0.51, SD = 1.33, median = 0). Only 25 (5.9%) participants had a score of 4 or more. The proportion of participants with a Knowledge scores of 0–6 varied by subgroup (ethnicity, gender, social class and age) (Table 2).

**Table 2:** Knowledge scores by ethnicity, social class; age and gender

		**KNOWLEDGE SCORE**							
		**0**	**1**	**2**	**3–4**	**5–6**	Mean score	SD	p-value*
**Ethnicity**									
White	N	235	30	13	13	14	0.61	1.416	
	%	77.0	9.8	4.3	4.3	4.6			
Non-White	N	105	4	1	2	3	0.26	1.035	0.001
	%	91.3	3.5	0.9	1.7	2.6			

**Social Class**									
Non-manual (I-IIIN)	N	128	22	8	12	15	0.90	1.745	
	%	69.2	11.9	4.3	6.5	8.1			
Manual (IIIM-V)	N	212	12	6	3	2	0.20	0.742	<0.001
	%	90.2	5.1	2.6	1.3	0.9			

**Age**									
16–24	N	96	8	3	2	1	0.25	0.826	
	%	87.3	7.3	2.7	1.8	0.9			
24–34	N	73	9	3	4	2	0.44	1.128	
	%	80.2	9.9	3.3	4.4	2.2			
34–44	N	104	7	4	5	6	0.52	1.390	
	%	82.5	5.6	3.2	4.0	4.8			
44–54	N	67	10	4	4	8	0.86	1.779	0.034
	%	72.0	10.8	4.3	4.3	8.6			

**Gender**									
Male	N	136	12	3	4	4	0.35	1.091	
	%	85.5	7.5	1.9	2.5	2.5			
Female	N	204	22	11	11	13	0.61	1.450	0.05
	%	78.2	8.4	4.2	4.2	5.0			

* derived from Kruskall-Wallis test for age; Mann-Whitney U test for other factors

Significant differences in knowledge scores were observed between ethnic groups (scores >0: 23% of white and 8.3% of non-white category); social class (scores >0: 30.1% of non-manual and 9.8% of manual category); with increasing age (scores >0: 16.24 = 12.7%, 25–34 = 19.8%, 35–44 = 17.5%, 45–54 = 28%), and by gender (scores >0: males = 14.5%, females = 21.8%) (Table 2). No differences in knowledge scores were revealed between parents and those who were not parents (mean scores = 0.58 and 0.40 respectively) (p = 0.285) or between the three geographical areas surveyed (p = 0.19).

Logistic regression modelling explored the effect of gender, age group, ethnic group and social class on knowledge scores (any knowledge vs. no knowledge). Predictors of having no knowledge were non white ethnicity (adjusted odd ratio = 2.96, 95%CI 1.44 to 6.10) and being a manual worker (OR = 4.10, 95%CI 2.39 to 7.06). Older age (45+ years) resulted in a decreased probability of no knowledge (OR = 0.32, 95%CI 0.30 to 0.96). After including ethnicity, social class and age band in the model, gender was no longer a significant factor.

### Attitude towards HPV Vaccination

Three hundred and seventy (88.1%, 95% CI 84.4% to 90.9%) participants agreed or strongly agreed with HPV vaccination, 6% had no opinion and 6% disagreed or strongly disagreed. The median score was 2.0 (1 = Strongly Agree, 5 = Strongly Disagree); participants tending to report that they agreed with vaccination. The mean response for attitude for the white ethnic group was 1.88 (Table 3) whereas, the mean response for the non-white group was 2.13; the white ethnic group were more inclined to agree with vaccination. This association was significant at the 5% confidence level by the Mann-Whitney U test (p = 0.006). (Table 3) Attitude did not differ significantly by social class, gender, parental status or between the three geographical areas surveyed.

**Table 3:** Attitude by ethnicity, social class; age and gender

		**ATTITUDE**							
		**1**	**2**	**3**	**4**	**5**	Mean score	SD	p-value*
**Ethnicity**									
White	N	80	197	15	11	2	1.88	0.71	
	%	26.2	64.6	4.9	3.6	0.7			
Non-White	N	21	72	10	10	2	2.13	0.87	0.006
	%	18.3	62.6	8.7	8.7	1.7			

**Social Class**									
Non-manual (I-IIIN)	N	41	123	9	10	2	1.97	0.77	
	%	22.2	66.5	4.9	5.4	1.1			
Manual (IIIM-V)	N	60	146	16	11	2	1.93	0.77	0.62
	%	25.5	62.1	6.8	4.7	0.9			

**Age**									
16–24	N	27	73	7	3	0	1.87	0.64	
	%	24.5	66.4	6.4	2.7	0.0			
24–34	N	26	55	4	6	0	1.89	0.77	
	%	28.6	60.4	4.4	6.6	0.0			
34–44	N	19	88	11	6	2	2.08	0.76	
	%	15.1	69.8	8.7	4.8	1.6			
44–54	N	29	53	3	6	2	1.91	0.89	0.047
	%	31.2	57.0	3.2	6.5	2.2			

**Gender**									
Male	N	38	104	11	3	3	1.92	0.74	
	%	23.9	65.4	6.9	1.9	1.9			
Female	N	63	165	14	18	1	1.96	0.78	0.769
	%	24.1	63.2	5.4	6.9	0.4			

* derived from Kruskall-Wallis test for age; Mann-Whitney U test for other factors.

ATTITUDE:

1 = STRONGLY AGREE.

2 = AGREE.

3 = NO OPINION.

4 = DISAGREE.

5 = STRONGLY DISAGREE.

Attitude assessed by the question: "Do you think that the introduction of HPV vaccination is a good idea?

Logistic regression modelling explored the effect of age group and ethnicity on attitude (positive attitude to vaccination vs. other). The only significant predictor of not having a positive attitude to vaccination was non- white ethnicity (odds ratio = 2.62, 95%CI 1.16 to 5.92).

### Other data

Participants were asked whether they were parents or not but this was not associated with either the knowledge score or attitude response.

A large proportion (70.5%) of the study population would want the vaccination to be free. The majority (59.3%) of participants thought that the proposed age group (10–12 years) was acceptable, whereas 34.3% disagreed.

The majority of study participants believed that boys should be vaccinated as well as girls (91.2%).

The majority of participants (83.6%), both parents and non-parents. reported that they would give consent for their child to have the vaccination.

## Discussion

This study assessed knowledge and attitudes towards HPV vaccination at a time before the UK Government announced its plans for a vaccination programme. Participants aged 16–54 were recruited from three different areas of Birmingham. The majority of participants had little awareness of HPV or HPV vaccination and 81% had a knowledge score of zero. Nevertheless, after the provision of brief HPV related information messages (Table 4), most (88%) participants were supportive of HPV vaccination. These findings are in accordance with previous UK-based studies which have demonstrated that people generally know little about HPV, but after the provision of information, most support the introduction of the vaccine [27-30] and re-enforce the need for more educational intervention in order to raise awareness [36].

**Table 4:** HPV related information provided to participants

• Most cervical cancer is caused by HPV infection.
• HPV is a sexually transmitted infection that can affects both males and females.
• The vaccine protects against HPV infection.
• The government is considering introducing the vaccination.
• Like rubella, the HPV vaccination will be a school based vaccination programme and be given to girls between 10 – 12 years.
• Vaccination only effective in people that have not yet been infected with HPV.

Although generally low, knowledge scores were higher in the white ethnic group than in the minority ethnic groups and in the non-manual worker group compared to the manual worker group. The white ethnic group were more in favour of HPV vaccination than the minority ethnic group. Both the social class groups were willing to accept HPV vaccination. Stepwise modelling selected social class in preference to the other explanatory variables in predicting attitudes towards HPV vaccination. There was a high degree of correlation between ethnicity, social class group and area of residence and each of these factors may be a surrogate for educational attainment.

However, a relatively large proportion of participants (34.3%) reported concern at the proposed target age group (10–12 years old). Most participants that disagreed with this target group reported that they felt the age group was too young. This survey was undertaken at when the likely age-group for vaccination was believed to be 10–12 years. The recent announcement that the target age group has been increased to 12–13 years may result in increased acceptability. This supports the findings of previous studies in both the USA [37] and UK [38] which have reported less parental support for vaccination in younger age groups.

The small group of the public (6%) who expressed no opinion towards the vaccination could be due to the fact that they had no or very little knowledge about the vaccination and didn't feel informed enough to form an opinion. Increased awareness and relating to vaccination and the reasons for vaccinating girls at age 12–13 years may address this.

The vast majority (83.6%) of participants (parents and non-parents) would consent for their child to be vaccinated. This implies a high uptake of the vaccination within the UK population, although reported action does not always reflect actual behaviour.

Limitations of this study include the possibility of sampling bias. As with most cross-sectional surveys, this street survey was only completed by a self-selected group of individuals who may not be representative of the general population. Similarly, the language barrier may have affected uptake ratio in the ethnic minority population, however, the vast majority of the people approached were fluent in English even if it was not their first language. It is probable that those not confident in written English were enabled to participate by the use of a semi-structured interview rather than a postal questionnaire.

Pilot work identified that the initial approach to the public was important; initial reactions often being that the interviewers were attempting to sell a product. Therefore, in the actual survey, all participants were approached by medical students who stated that this was a University research project.

People who failed to take part in the study might differ in their attitudes towards HPV vaccination. Unfortunately, due to the nature of the street survey (purposive sampling) refusal rates were not collected and it was not possible to establish the characteristics of non-participants. It is probable that those with no knowledge of HPV would be less inclined to participate. Knowledge of HPV and the proposed vaccination programme may therefore be over-estimated and social desirability bias may mean that reported acceptability of vaccination is over-reported.

This study involved researcher/participant discussion about a Sexually Transmitted Infection (STI). STI's are a sensitive subject especially within different cultures. Questioning people's attitudes towards HPV could be potentially embarrassing for the participant and may have deterred honest responses. To minimise the embarrassment experienced by participants a very simple knowledge score comprising only six questions was used; this may not have been as sensitive as more detailed surveys [25].

This study supports the findings of previous studies in respect to the public's knowledge and attitudes to HPV and the vaccination. The majority of the existing research has assessed the attitudes and knowledge of adolescents and their parents, especially mothers [16-18]. This survey has demonstrated that, in the UK, the attitudes and knowledge of parents and non-parents are similar and it therefore may be possible to generalise from studies focussing on parents to the wider population.

## Conclusion

The study confirms that there is still a lack of information or access to information about HPV and that more needs to be done to raise awareness of HPV and HPV vaccination especially amongst the minority ethnic groups and the lower social classes. Nevertheless, if the vaccination is introduced it appears that a significant number of people would be agreeable to vaccination after the provision of the quite simple messages.

There is a need for the health care service and other agencies to play a more active role in publicising, educating and informing patients on HPV and potential value of HPV vaccination.

Further work is required to look at the attitudes of health professionals in the UK towards HPV vaccination.

In conclusion, the success of the newly proposed HPV vaccination will depend on public attitudes and acceptance. This study demonstrates that most people state that they would accept the vaccination if it were offered, but more information is needed to increase knowledge. Despite the general view being positive, many people expressed a wish to know more about vaccination. Possible side effects, efficacy, costs and the age group were highlighted as areas of concern.

## Competing interests

The authors declare that they have no competing interests.

## Authors' contributions

CDW wrote the first draft of the introduction and literature review, AG and AS the first draft of the methods and MS, JP and SW undertook the analyses. Abstract, results, discussion and other sections were equally contributed to by all medical students. SW and JP supervised the project. SW had the idea for the study, and is the guarantor. All the authors contributed to the final version of the manuscript and have approved this version.

## Pre-publication history

The pre-publication history for this paper can be accessed here:


